# Controllable Crimpness of Animal Hairs via Water-Stimulated Shape Fixation for Regulation of Thermal Insulation

**DOI:** 10.3390/polym11010172

**Published:** 2019-01-18

**Authors:** Xueliang Xiao, Yanjia Gu, Guanzheng Wu, Diantang Zhang, Huizhen Ke

**Affiliations:** 1 Key laboratory of Eco-textiles, Ministry of Education, Jiangnan University, Wuxi 214122, China; xiao_xueliang@jiangnan.edu.cn (X.X.); yanjiagu1984@163.com (Y.G.); guanzhengwu@163.com (G.W.); zhangdt787@126.com (D.Z.); 2 Fujian Key Laboratory of Novel Functional Fibers and Materials, Minjiang University, Fuzhou 350108, China

**Keywords:** animal hairs, crimpness, temporary shape fixation, thermal insulation

## Abstract

Animals living in extremely cold plateau areas have shown amazing ability to maintain their bodies warmth, a benefit of their hair’s unique structures and crimps. Investigation of hair crimps using a water-stimulated shape fixation effect would control the hair’s crimpness with a specific wetting-drying process thereafter, in order to achieve the regulation of hair thermal insulation. The mechanism of hair’s temporary shape fixation was revealed through FTIR and XRD characterizations for switching on and off the hydrogen bonds between macromolecules via penetration into and removal of aqueous molecules. The thermal insulation of hairs was regulated by managing the hair temporary crimps, that is, through managing the multiple reflectance of infrared light by hair hierarchical crimps from hair root to head.

## 1. Introduction

Animal hair fibers have been traditionally considered for producing good thermal insulation of textiles for thousands of years. The typical case is the wool fiber. Jun et al. [1] stated that a dense pack of wools would form a stable air layer that affects a human’s thermal sensation and thermal comfort when wools are in the middle of skin and textiles, indicating the wool’s role in good thermal insulation. However, a limit of wool textiles in developing super thermal insulation results in the appearance of feather down jackets [2,3] and, later, the hot research of aerogels [4,5] as an outstanding thermal barrier in the application of aerogel products in harsh conditions. Their features manifest through the high porosity constructed by the specific fractal structure in nature or man-made foaming technology. Besides, until recently, a biomimetic study had set off a few novel processes for developing ordered porous structure of materials for higher thermal insulation. For example, Bai et al. [6] developed an ordered porous silk filament using a self-developed “freeze-spinning” method by mimicking the directional porous structure of polar bear’s hair. Such methods have inspired biomimetic aerogel [7] from polar bear hair, core-sheath structure of phase change nanofibers using nano-encapsulation technology [8], and electrospinning [9] to be developed for higher thermal insulation materials. The directional ordered porous structures of bio-inspired filaments or nanofibers were reported to benefit the improvement of thermal insulation sufficiently.

**Figure 1:**
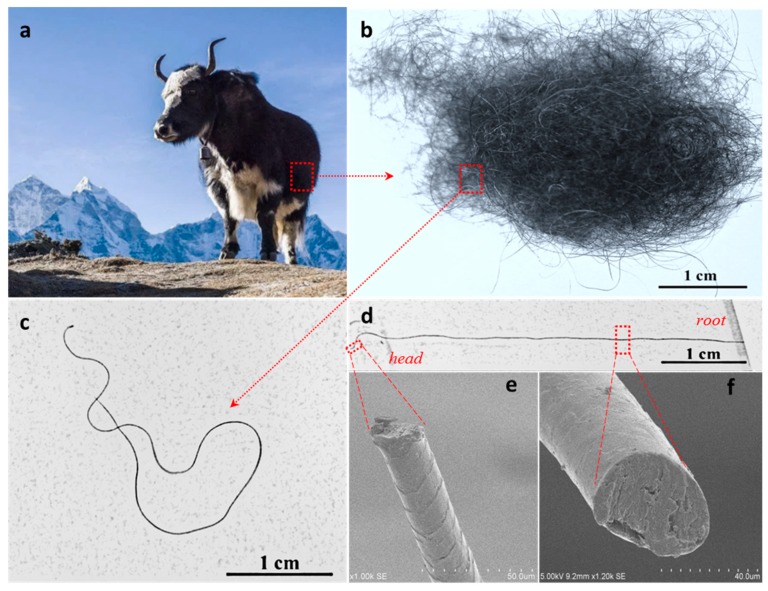
Yak living in a cold plateau area with thick, warm fur and hair layers, (**a**) photograph of a yak living at Tibet plateau of more than 4000 m above sea level, (**b**) raw yak hairs with guard and down hairs, (**c**) a yak hair with natural crimps, (**d**) the stretched yak hair with more times in length than natural length, (**e**) cross section of head part of hair, and (**f**) root coarse part of hair.

Feather and aerogel belong to high porosity and ordered porous structure of warmth retention materials. However, some other animals have thermal insulated materials with unique structural features to keep their bodies warm. For instance, some mammals living in extremely cold areas have displayed unbelievable abilities to survive [10,11]. For example, antelope and yak use their thick fat fur covered by crimped hairs to effectively absorb and reflect infrared emission from their bodies, making them even invisible under an infrared camera [12,13,14,15]. In contrast to polar bears, yak living at more than 4000 m of a cold plateau area (Figure 1a) are covered with thick, long hairs with a large number of crimps to keep their bodies warm, as shown in Figure 1b,c. A yak’s black fur and hairs absorbs visible and infrared light from the sun that warms the body; on the other hand, this can prevent the heat emission from the inside body [16]. Figure 1b also indicates that the raw yak hairs with multiscale structure, in which the coarse hair is surrounded with many fine hairs, have an increased number of crimps. A single strand of yak hair also shows various natural spiral crimps (Figure 1c) that can be stretched to a straight line (Figure 1d) with two constrained heads. The stretched yak hair shows an ability to recover its original spiral crimps, much like a spring, when the constrain of the two heads is released. The fine and coarse parts (Figure 1e,f) of the yak hair both display solid cross sections without porous medulla, unlike a polar bear’s hollow hair with its internal regular porous structure, indicating the high significance of hair crimps in its comprehensive warmth retention for yak who live in cold plateaus. Besides, the well-proportioned coarse and fine hairs on the surface of a yak’s body also allow the yak to survive in harsh Tibetan plateau regions [17]. For example, the long and coarse hairs are normally on the outside surface of the yak body to provide protection or a warmth barrier, while the fine and cashmere hairs with a large number of crimps are present beneath the coarse and straight hairs to better cut off warmth emission from inside its body.

Here, it is intriguing to investigate the contribution of hair crimps to thermal insulation, as well as the underlying mechanism. Understanding of the feasibility to control the hair crimps and the effect of hair crimping variation on the thermal insulation is essential for further mimicking of such structural biomaterials in the synthetic world. In this study, we report a controllable approach to hair crimps for regulation of hair thermal insulation using the shape memory function of hair stimulated by water. The related shape fixation mechanism and the changed heat retention were also studied. The corresponding experimental materials and methods are supplied in the Experimental Section.

## 2. Experimental Methods

### 2.1. Materials

Yak hairs in the raw state were purchased from Hongsheng Yakwool Co., Ltd., Qinghe, China. The hair samples were firstly combed to remove impurities between hairs. Then, the samples were soaked in an ethanol or chloroform solution for one minute to clear the hair of fatty materials [18]. The soaking process of the yak hairs was assumed to have negligible effect on the hair crimpness and strength. In contrast, after removal of the fatty film, the hairs interact more efficiently with water for temporary shape fixation. The fresh samples were obtained after two rinses in distilled water and one hour of the drying process at 40–60 °C in an oven. The hair samples were tested for specific moisture regain at the range of 12–14%. It was also statistical to the purchased hair diameter with a discrepancy of 15–25 μm (30%), 25–45 μm (55%), and >45 μm (15%) through counting the number of hairs after measuring their diameters at each hair’s middle part. The lengths of the purchased hair samples (coarse and fine hairs) were also different after the measurements, usually, 1–2.5 cm for diameter of 15–25 μm, 2.5–5 cm for diameter of 25–45 μm, and >5 cm for diameter of >45 μm, respectively.

### 2.2. Shape Fixation Temporarily for Controlling Crimpness

The crimped yak hairs were immersed in water at room temperature for half a day to ensure full interaction of the hair cortex with the water. In water, the hair samples were manually wrapped on a flat bar and maintained their shape for one hour to endow the hairs with full plasticization. The fixed hairs were then taken out of the water and dried for one day. When the temporarily fixed, entangled dry hairs encountered water, the shape recovery to original crimps took place. This shape memory program of the yak hair stimulated by water can also refer to the methods of our previous works (camel hairs) [18,19] that the stimulus (e.g., water, reducing solution, etc.) should fully interact with the deformed hair for complete release of the locked switch, and a reverse process takes into account the lock of the deformed hair for temporary shape fixation. Here, it only takes half of the shape memory function to control the hair crimps for shape fixation and, in each process, the hair shape was observed using an optical camera.

### 2.3. Characterization of Hair Shape Change

The yak hair with crimps were measured for the diameter (the middle part of the hair) and the number of semi-crimps along the fiber axis under a microscope with low magnification. In order to measure the relationship of hair crimpness and diameter, 18 kinds of diameter of yak hairs were selected for the determination and, for each diameter, five hairs were measured for average values and standard deviations. The same number of yak hairs were also used to measure the shape fixation ratios by water. The cross-section yak hairs were cut using a sharp blade. Then, the hair cross section was gold-coated and observed using an environmental scanning electron microscope (SEM, JEOL Model JSM-6490, Tokyo, Japan). Over the hair shape fixation and recovery, the chemical groups and hydrogen bonds were examined using Fourier Transform Infrared Spectroscopy (PerkinElmer Spectrum 100 FT-IR Spectrometer, Waltham, MA, USA) in the scan range of wave numbers from 4000–650 cm^−1^ using the ATR (Attenuated-Total-Reflectance) method. The absorption spectra were recorded with eight scans at a resolution of 8 cm^−1^. The angle (ϕ) of incidence light was adjusted to 40°, the ATR crystal was diamond (refractive index *n*_1_ is 2.4), and the refractive index of the hair fiber (*n*_2_) was around 1.5. The characterized depth of penetration ($d_{p}$) was in the range of 1–15 μm. The crystalline phase of the hair in dry and wet states can be characterized by X-ray diffraction due to the Bragg regular arrangement, which is called the Rigaku Smart Lab XRD system (9KW, Rigaku Corporation, Tokyo, Japan) and is equipped with Cu Kα radiation with a wavelength of 1.54 $\dot{A}$. Before scanning, the hairs were minced in the format of short chips to cover the stage. The test 2θ range is from 5° to 30° and recorded at a scan speed of 10°∙min^−1^ at 40 kV and 40 mA. In respect of reflectance (containing absorption, unit as %) measurement of visible to near infrared light, the dry hair samples at the same weight were observed by an infrared-visible spectrophotometer (U-4100, HITACHI, Tokyo, Japan). The diameter of the fine hair sample was in the range of 15 to 30 μm, while the coarse hair was from 30 to 50 μm. The test wavelength was set from 450 to 800 nm, and the resolution was set as 10 nm per scan. To measure the thermal insulation properties, all infrared images for different hair states were taken by a thermal image camera (FLUR-E_5_, FLIR Systems, Boston, MA, USA). The working distance was ≈30 cm.

## 3. Results and Discussions

### 3.1. Physical Properties of Crimped Yak Hairs

**Figure 2:**
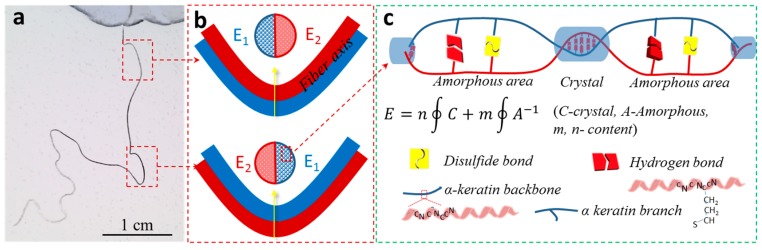
How does the hair crimp? (**a**) A yak hair in free crimped state, (**b**) a crimp made of orthocortex and pi-cortical cells with different modulus E_1_ and E_2_ along the hair axis, and (**c**) in either cortex made of different contents of crystalline and amorphous phases for the hair crimping.

The crimps of any α-keratin hair are normally generated during the growing of cortex ortho and para cells. These two kinds of cells showed an unbalanced modulus along the fiber central axis, resulting in many irregular crimping circles in space, as shown in Figure 2a.

For any crimp, the ideal hair cross section showed two parts with the inequivalent modulus (E_1_ ≠ E_2_) or rigidity where para- and ortho-cortex components appeared on the outside of the crimp alternately to form natural crimps, as shown in Figure 2b. The proportions of crystalline and amorphous phases were different in both parts where the higher rigidity part contained more crystalline phases, as shown in Equation (1) in Figure 2c:

(1) $$E=n\oint C+m\oint A^{-1}.$$

Here, E is the modulus of either cortex component, C stands for the crystalline phase, and A represents the amorphous phase, with *n* and *m* denote the contents of either phase at any hair cross section. Equation (1) indicated that more contents of the crystalline phase would give rise to a higher modulus. The amorphous area was mainly made of microfibrils or keratin macromolecules with large space between each other, using branch molecules or hydrogen bonds to connect them (Figure 2c), while the crystalline phase was composed of regular aligned microfibrils with dense hydrogen bonds connecting macromolecules in fibrils. The growing rate of the crystalline phase was less than the softer amorphous keratin cells, therefore, the looser components were growing longer than the dense cortex in length for the inside directional crimp. Thus, the dense and loose components of either cortex provided opportunities for controlling the crimping using shape memory theory. The dense crystalline phase may have played the role of netpoint, while the soft amorphous area may have been the switch for shape temporary fixation and recovery.

Here, the length of two hair ends defined the hair original length (L_o_). The ratio of the stretched hair length (L_s_) to its original length defined the hair crimpness [20]. The higher value of the ratio indicated a higher number of hair crimps at the same hair diameter, which should have had a better heat retention. Thus, it was interesting to find out the relationship of crimpness and hair diameter that could guide the controlling of hair crimping effectively.

**Figure 3:**
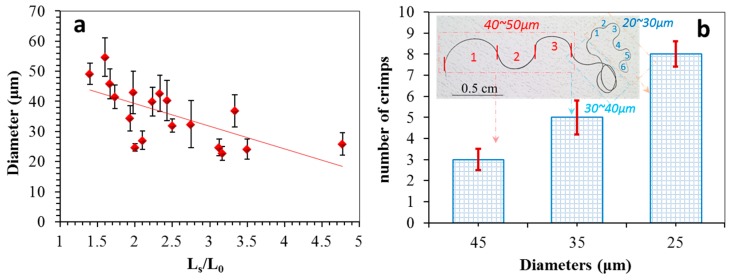
Physical properties of yak hairs. (**a**) Measured relationship of hair diameter and ratio of stretched hair length (L_s_) to natural crimped hair length (L_o_). (**b**) Distribution of crimp numbers along a mature yak hair from its coarse to fine parts.

In comparing the crimpness of different hairs, Figure 3a reflects the measured crimpness related to the hair diameter (18 × 5 samples), in which most values were in the range of 1.5 to 3.5, corresponding to the diameter range of 20 to 40 μm. Normally, the diameters of natural yak hairs are in the range of 15 to 80 μm. Experience tells us that crimpness decreases with the increase of hair diameter [20,21,22,23]. Conversely, Figure 3a echoes this trend in showing the measured increase in crimpness with the decrease of hair diameter. On the other hand, to compare the number of crimps for each yak hair, the number of semicircles was counted from its root to head under different diameter ranges; for instance, the hair has three semicircles for the diameter range of 40–50 μm, as shown in the count on the inset image of Figure 3b. The histogram indicates that the finer part of the hair had more crimps under the unit length. This indicates that the finer hair also had a greater number of crimps. For a coarse hair, especially with diameter beyond 60 μm, the hair would seldom have crimps, i.e., guard hair, reflecting a kind of protection to the inside down fibers [24].

**Figure 4:**
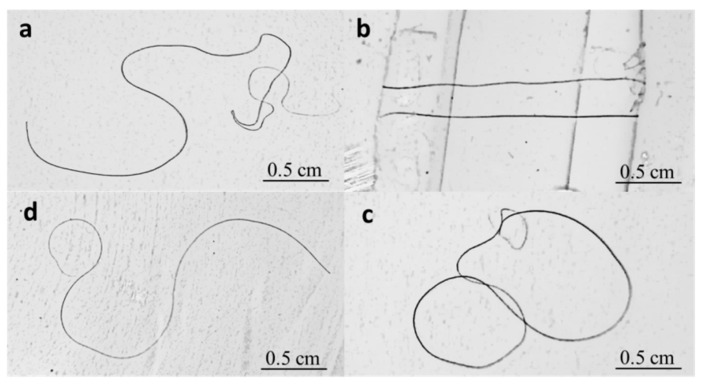
Shape memory effect of yak hair responsive to water, in which (**a**) shows a natural crimped hair, (**b**) denotes the manually deformed yak hair soaked in water, (**c**) displays the temporarily deformed shape of hair after a drying process, and (**d**) shows the recovered crimped shape of hair stimulated by water.

With finding of the relationship of hair crimpness and diameter, is there any possibility to improve the crimpness of coarse hairs manually? The natural crimps with high warmth retention are significantly important to yaks and other animals in extremely cold regions. In reference to Figure 2, as a kind of α-keratin fiber, the shape of hair crimps may be controlled using shape memory [18,25] or stress memory [26] functions. Here, a shape memory program of a typical yak hair responsive to water was carried out to determine the controllability of hair crimps, as shown in Figure 4. The key steps of Figure 4b,c show the approaches in making a temporary crimp shape. The step Figure 4d shows is the recovered shape that can be compared with the original shape. 

**Figure 5:**
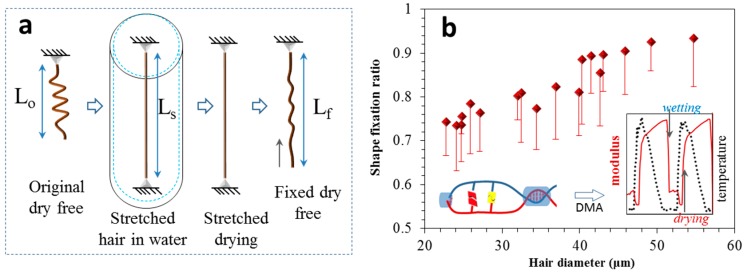
A quantitative shape fixation test of yak hair, (**a**) test principle with four steps (original length (L_o_)→length of stretched hair in water (L_s_)→fixed length of hair after drying process (L_f_)), (**b**) the measured shape fixation ratios related to hair diameter, where the inset refers to our previous publication [18] that wetting can reduce the hair storage modulus by opening the hydrogen bonds in hair amorphous area and drying process shows the reverse function. The inset figure in (**b**) is reproduced from [18] under open access license.

Theoretically, the wetting and drying processes relative to the hair crimps can switch the reversible hydrogen bonds inside the hair polypeptide chains on and off, respectively [18], giving rise to the controllable dry shape temporarily as designed, such as particular crimps with specific semicircle diameters or designed spiral angles. However, experimental data showed that the temporary fixed crimps were not completely the same as designed, but also a hundred percent different with the original shapes. A shape fixation ratio should be investigated to ensure smart controllability [25]. Figure 5 shows a quantitative investigation of shape fixation ability ($R_{f}$, Equation (2)) of yak hair using water as stimulus.

Figure 5a shows the shape fixation program, and Figure 5b shows the test results. The shape fixation ratio ($R_{f}$) was obtained according to Equation (2), based on the measured lengths of original, stretched, and fixed states of hairs, where

(2) $$R_{f}=\frac{L_{f}-L_{o}}{L_{s}-L_{o}}.$$

With the increase of hair diameter, the hair shape fixation showed an increased ability that may be ascribed to the higher amount of hydrogen bonds in the amorphous area that can lock the temporary shape more easily during the drying process. The inset image of Figure 5b indicates a DMA test of a keratin hair where the wetting process could reduce the hair modulus, while the drying process would take a reverse role. The wetting and drying processes endow the hair with a periodical up and down cycle of modulus, indicating the reversible controllability of yak hair in shape or crimpness. However, it must be pointed out that the shape fixation ratio at around 0.8 for most yak hairs (20–40 μm in diameter) revealed the highest ability of water as a stimulus in crimpness controlling. This means a crimp range may be achieved beyond the crimp that was set before processing.

### 3.2. Effect of Manual Shape Fixation on Thermal Insulation of Yak Hairs

**Figure 6:**
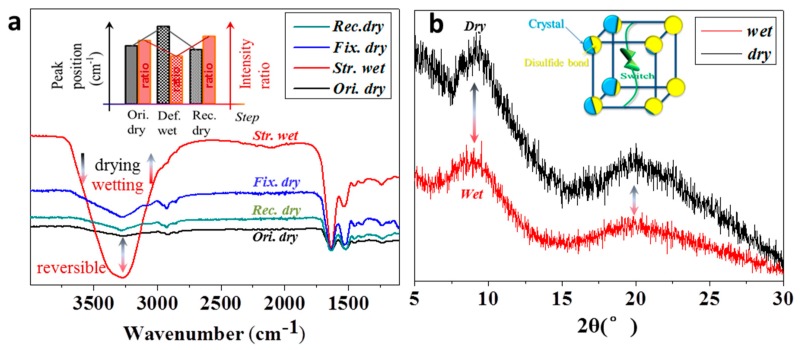
Reversible hair shape fixation and recovery using characterizations from (**a**) Fourier transform infrared (FTIR) (the inset figure shows the reversible feature of hair shape memory responsive to water in terms of IR peak intensity and abscissa position, reproduced from [18] under open access license) and (**b**) X-ray diffraction (XRD) (the inset figure indicates the twin-netpoints for intact hair shape in shape memory, reproduced from [19] by permission of The Royal Society of Chemistry) for hairs in dry and wet states.

In Figure 5, the “stretched hair in water” experienced the decline of hair modulus, indicating the opening of original hydrogen bonds for later establishing a new stretched hair shape, while the “stretched drying” means the constrained hair generated new hydrogen bonds during drying for fixing the hair in a temporary shape. The reversible variation of hair modulus under wetting and drying processes can be reflected through the changes of hydrogen bonds by watching the characteristic IR peaks from their peak abscissa and intensity ratio [18,19,27], as shown the inset image in Figure 6a.

In detail, the wetting process introduced free water into the hair sample that resulted in the IR curve with a highly increased intensity absorption band at around 3400 cm^−1^ [28]. The change of intensity ratio between two characteristic peaks of stretching C=O (Amide band I, 1629 cm^−1^) and bending N–H (Amide band II, 1531 cm^−1^) vibrations were found with the decrease and increase from the hair states of dry (Ori. dry) to wet (Def. wet) and wet (Def. wet) to dry (Fix. dry) or wet (Rec. wet) to dry (Rec. dry) reverses, respectively. The intensities of both characteristic peaks at Amide bands I and II almost equaled each other for the dry hair, while the decreased peak intensity ratio of the wet hair means the penetrated aqueous molecules disrupted the hydrogen bond formed between both bands, resulting in the lower number of band II of 1510–1535 cm^−1^ and the consequently reduced intensity ratio [29,30]. In respect to wavenumber shifting, the characteristic peaks of both bands were shifted from 1625 to 1629 cm^−1^ and 1515 to 1531 cm^−1^, respectively, corresponding to the dry hair in the hydration process. In turn, the wavenumber is decreased by 4 to 8 cm^−1^ from wet to dry states, relative to the drying process. This reversible shifting corresponding to the conversion between drying and wetting conditions suggests that the intermolecular hydrogen bonds underwent the reversible destruction and formation processes, accordingly [31]. In summary, the inset of Figure 3a indicated that the hair shapes in original dry state (Ori. dry), deformed wet state (Def. wet), and recovered dry state (Rec. dry) can be converted to each other, judging from their IR characteristic peaks in shifting of wavenumber and variation of peak intensity ratio. This indicates the switch role of hydrogen bond for controlling the hair crimping shape [32].

In Figure 6b, the characteristic bands at 9.2° and 21° represent the particular α and β types of crystalline phases formed by intense hydrogen bonds between strongly regular polypeptide chains [33]. Pioneers reported that the keratin crystals are hard to be cleaved by polar molecules under room temperature [34,35]. The evident peak intensities of both curves testified the intact crystalline phase during the wetting and drying process of the yak hair. The slightly low intensity of wet yak hair may be ascribed to the margin of crystalline phase influenced by free aqueous molecules in polarizing the intense hydrogen bonds. Nevertheless, the inset of Figure 6b has summarized a structural model for explaining the water induced shape memory behavior of α-keratin hair, i.e., the twin-netpoint and single switch model [18,19]. The model benefitted the design of temporary shapes of hair, especially the hair crimps. It is believed that the re-defined hair crimps in various geometrical shapes could adjust the heat retention property, realizing a function of the hair with controllable shapes and thermal properties, through which to biomimic some particular synthetic filaments with controllable thermal behaviors.

**Figure 7:**
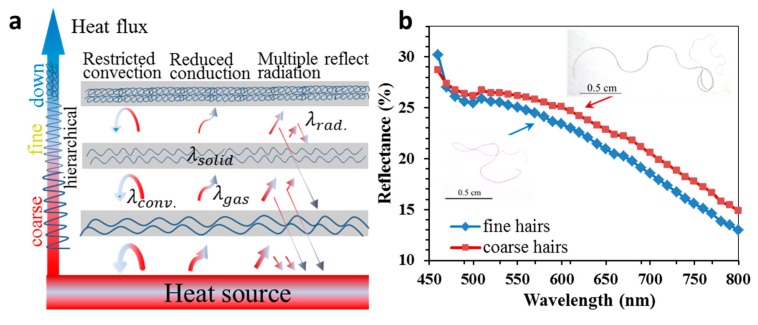
Thermal insulation of yak hairs. (**a**) Schematic illustration of thermal conductivity, convection, and radiation of yak hairs from coarse to fine crimps; (**b**) the reflectance (including absorption) measurement of visible to near infrared lights for coarse (diameter = 40 to 60 μm) and fine (diameter = 20 to 40 μm) yak hairs, using a visible- infrared spectroscopy microscope.

Figure 7a illustrates the heat transfer through a mass of yak hairs with a large number of hierarchical crimps. The ‘hierarchical’ here means the hair crimp diameter (crimp circle) and density (number of crimps per unit length) was decreased gradually in similarity to the real hierarchy of one by one order of magnitude. This crimp structure had been optimized by nature for the highest ability to improve the reflectance of infrared light with numerous solid–air interfaces in the hierarchy [36]. Here, for any coarse and fine yak hairs, we assumed the thermal conductivity was the same for the cortex fibrils ($\lambda_{solid}$, main body of hairs). Theoretically, the thermal conductivity of a mass of crimped hairs ($\lambda_{cf}$) was determined by the sum of thermal convection ($\lambda_{conv}$), fiber ($\lambda_{solid}$) and air ($\lambda_{air}$) thermal conduction, and thermal radiation ($\lambda_{rad}$) [37,38]:

(3) $$\lambda_{cf}=\lambda_{conv}+\lambda_{solid}+\lambda_{air}+\lambda_{rad}.$$

Thermal convection of the crimped hairs was highly restricted as air was blocked within hierarchical microgaps divided by various coarse, fine, and down hairs. As the mass of crimped fibers displayed high porosity, and air had much smaller thermal conduction than fiber body, the sum of thermal conduction was significantly reduced. Besides, the divided microgaps contributed to the low thermal conduction, as the microgap size was close to the mean-free path of the gas molecules in the stored air. Furthermore, heat was radiated mainly by the invisible infrared light, and the ‘hierarchical’ aligned microgaps from the skin surface to outside, which were considered to be more suitable for reflectance than random alignment, could have largely improved the infrared reflectance. The emission intensity of radiation from a heat source is given by Wien’s displacement law [39], where

(4) $$\lambda_{rad}{}^{\prime }=\frac{b}{T}.$$

Here, $\lambda_{rad}{}^{\prime }$ represents the mean wavelength of the infrared light for thermal radiation, and b is the constant of 2900 μm·K. The calculated wavelength of the infrared light is 9.5 μm if the temperature of human body surface is assumed to be 306 K (33 °C). This wavelength and hair crimp diameter show the same order of magnitude (5–25 μm) due to resonance, indicating the highest barrier of hair crimps to infrared light emission for high thermal insulation. Here, Figure 7a illustrates a multiple reflective effect by hierarchically aligned hair–air incident angle. After the multilayer of reflectance from skin to outside, the heat intensity of infrared light reduced significantly. Figure 7b shows the measured reflectance of visible to near infrared lights by crimped hairs for comparing them in terms of hair diameter and crimpness. It was noted that both coarse and fine crimped yak hairs displayed a descending trend of reflectance with the increase of wavelength. This indicated that the visible light was easily reflected and absorbed by the dark color of the yak hairs, and infrared light with larger wavelength was prone to transfer through the thin layer of hairs. However, the involvement of the coarse root of hairs effectively increased the reflected/absorbed area for higher reflectance; on the other hand, the ‘hierarchical’ oriented crimps of hairs from hair root to head also manifested higher reflectance. This is the explanation for the reasonable existence of coarse hairs for the maximum of thermal insulation other than pure down hairs on the yak body surface.

**Figure 8:**
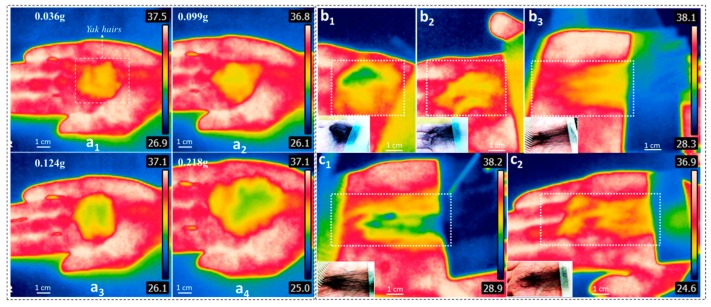
Thermal insulation properties of yak hairs with infrared images for (**a**) the measured relationship of hair weight (a_1_-0.036 g; a_2_-0.099 g; a_3_-0.124 g; a_4_-0.218 g) and temperature contour of hairs on a human hand (a kind of constant temperature source); (**b**) thermal insulation corresponds to hair states that (b_1_) shows hairs rubbed into a ball shape, (b_2_) shows hairs in natural state, and (b_3_) shows hairs in fully stretched straight state; (**c**) the fully stretched hairs in wet (c_1_) and dry (c_2_) states, using shape memory function.

In respect to characterization of hair thermal insulations, Figure 8 gives the measured infrared images by an infrared image camera for judging the thermal conductivity [40] of various yak hairs. In detail, Figure 8a illustrates the crimped yak hairs in various weights (a_1_~a_4_) for heat transfer. In comparison to the temperature contours of the measured hairs, it was noted that the thicker hairs resulted in less transferred infrared lights with lower observed temperature. This is the reason that yak living on the colder plateau areas are covered with thicker layers of hair. However, if we controlled the orientation of a certain mass of hairs, as shown of the inset images in Figure 8b_1_–b_3_, the transferred infrared lights would vary, depending on the hair crimp shape and alignment in the group. The compared images indicate that the stretched hairs without crimps showed greatly reduced thermal reflectance, giving rise to the most heat emission in the form of infrared light passing through the hair layer. Figure 4 and Figure 5 indicated the shape fixation and recovery ability of keratin hairs responsive to water, thus, a mass of stretched yak hairs was generated based on the temporary shape fixation process. Figure 8c_1_ shows the infrared image of wet, stretched yak hairs on a constant temperature source, such as a human hand (assumed surface temperature at 33 °C), where a high thermal insulation of such hairs were observed due to the heat absorption by aqueous molecules inside the hairs. However, after a drying process to the wet stretched hairs, the dry stretched hairs were investigated for thermal insulation. As shown in the stretched dry hairs in free status in Figure 8c_2_, the hairs with greatly reduced crimps manifested highly reduced thermal insulation performance in comparison to the hairs in natural crimped states, like Figure 8b_2_. This indicates the significance of hair crimps in thermal insulation, and in controllable thermal insulation of hair using a shape fixation function responsive to a specific stimulus. The thermal insulation of hairs in different states can be found in the supplementary file (Figure S1). This can contribute to the improvement of thermal insulation of some winter clothing using a temporary shape fixation program.

## 4. Conclusions

In summary, apart from hair’s hollow structure, the crimping structure of hair plays a key role in thermal insulation for some mammal animals, like yak, living in cold plateau areas. The yak hair (a α-keratin polymer) was found to have a shape memory function responsive to water that implies it is a controllable shape (temporary shape fixation) for its crimp optimization and modification of heat retention, also with inspiration to biomimicking of crimped synthetic filaments. Experimental data indicated that the wetting and drying processes gave the hair crimps various temporary fixed shapes using the hydrogen bonds in opening and relocking as a switch, while the hair maintained intact for the well-preserved crystalline phase as the netpoint. The infrared images showed that the temporarily fixed shapes of unconstrained hairs, fabricated using its water-induced temporary shape fixation function, indeed manifested different thermal insulations in comparison to the original crimped shapes of hairs.

## Supplementary Materials

The following are available online at http://www.mdpi.com/2073-4360/11/1/172/s1, Figure S1: Thermal insulation of hairs in different states.
