# Supplementary material for: Controllable Crimpness of Animal Hairs via Water-Stimulated Shape Fixation for Regulation of Thermal Insulation
Source: Polymers (Basel). 2019 Jan 18;11(1):172. doi: 10.3390/polym11010172 (PMC6401684; doi:10.3390/polym11010172)
Supplement: Supplementary file 1 [file polymers-11-00172-s001.pdf]

Thermal insulation of hairs in different states

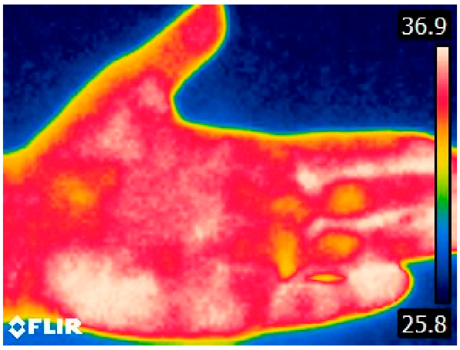

Pure hand

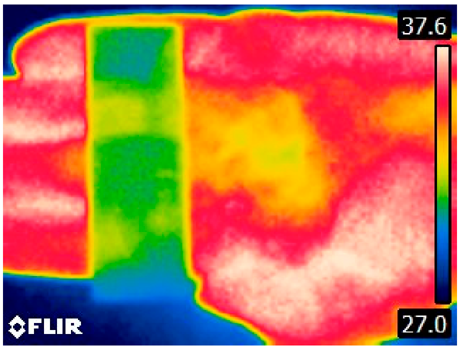

Natural original yak hairs

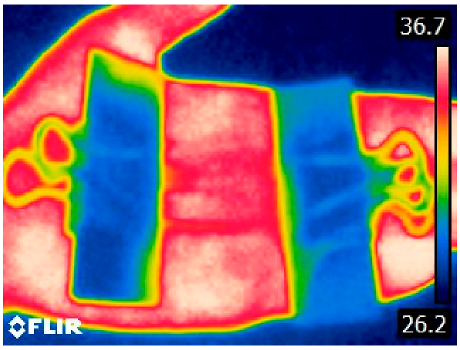

Stretched dry hairs

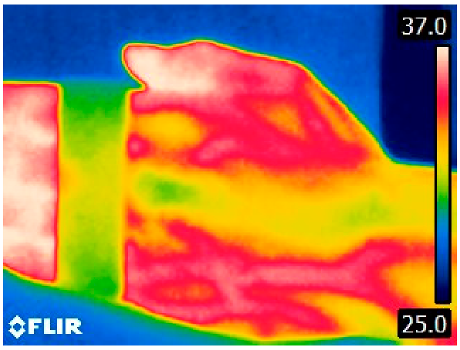

Shape fixed hairs
